# Thrombospondin-1 serum levels do not correlate with pelvic pain in patients with ovarian endometriosis

**DOI:** 10.1186/1757-2215-2-18

**Published:** 2009-11-16

**Authors:** Manuel García Manero, Begoña Olartecoechea, Pedro Royo, Juan Luis Alcázar

**Affiliations:** 1 Department of Obstetrics and Gynecology, Clínica Universitaria de Navarra, University of Navarra, Pamplona, Spain; 2 Department of Obstetrics and Gynecology, Hospital San Jorge, Huesca, Spain

## Abstract

**Objetive:**

Thrombospondin-1 serum levels is correlate with pelvic pain in patients with ovarian endometriosis.

**Patients:**

Thrombospondin-1 serum levels were prospectively analysed in 51 patients (group A asymptomatic patients or patients presenting mild dysmenorrhea and women comprised group B severe dysmenorrhea and/or chronic pelvic pain and/or dyspareunia) who underwent surgery for cystic ovarian endometriosis to asses whether a correlation exists among thrombospondin-1 serum levels and pelvic pain.

**Results:**

From 56 patients, five cases were ultimateley excluded, because the histological diagnosis was other than cystic ovarian endometriosis (2 teratomas and 3 haemorragic cysts). The mean thrombospondin-1 serum levels in group A was 256,69 pg/ml_+37,07 and in group B was 291,41 pg/ml + 35,59.

**Conclusion:**

Pain symptoms in ovarian endometriosis is not correlated with thrombospondin-1 serum levels.

## Introduction

Endometriosis is a common gynaecologic disease of unknown aetiology. The most widely accepted hypothesis for the development of endometriosis is retrograde menstruation. However, some other factor renders certain women susceptible to the implantation and growth of this ectopic endometrium.

Angiogenesis appears as one of the processes involved in the pathogenesis of endometriosis [1,2]. Angiogenic factors are increased in the peritoneal fluid of patients with endometriosis [3,4] in peritoneal implants [5] and in ovarian endometriomas[6,7].

On the other hand some investigators have found that angiogenesis is related to pelvic pain [8]. We speculated that ovarian endometriomas in patients presenting with pelvic pain would have more angiogenesis than those in asymptomatic women and, therefore, their vascular features would be different [9]. Previosly, we studied angiogenic factors (VEGF, IL-8) and their relationship with pelvic pain and conclude that these angiogenic factors not correlate with pelvic pain in ovarian endometriosis [10-13].

Angiogenesis is under the control of numerous inducers, including the vascular endothelial growth factor (VEGF) family and inhibitors, such as thrombospondin-1 (TSP-1) [9]

The aim of our study was to further investigate thrombospondin-1 serum levels in asymptomatic patients and women with pelvic pain to determine whether this antiangiogenic factor can be used as a serum marker of endometriosis activity.

## Patients

### Materials and methods

In this prospective study 56 pre-menopausal women (mean age: 34.38 ± 7.07) were enrolled from February 2003 to February 2005. Patients were divided in two groups according to clinical complaints. Group A included asymptomatic patients or patients presenting mild or moderate dysmenorrhea, but without dispareunia or chronic pelvic pain (n = 25) Group B included patients presenting severe dysmenorrhea (with no response to conventional analgesic, treatment such as antiprostaglandins and requiring bed rest) and/or dyspareunia and/or chronic pelvic pain. (n = 26). The degree of pain was established using a visual analogue scale, VAS scale [14].

All patients provided informed consent after the nature of the study was fully explained and Institutional Review Board approval (Clinica Universitaria de Navarra) was obtained before starting the study.

Blood samples were collected from all patients before anaesthesia by venipuncture into 10 cc sterile tubes and were kept at room temperature until centrifugation at 400 × *g *for 10 minutes. Less than 2 hours were allowed between blood collection and processing. Serum aliquots were then frozen at -80°C until measurement of thrombospondin-1 serum levels.

Serum concentrations of thrombospondin-1 were measured with use of an immunoassay (Quantikine; R&D Systems Inc., Minneapolis, MN). Thrombospondin-1 concentration can be measured in the range of 3.5 to 2,000 pg/mL. Interassay and intra-assay coefficients of variation were <10%.

### Statistical analysis

Statistical analysis was performed using the SPSS version 11.0 software (SPSS, Inc., Chicago IL). The mean serum level of thrombospondin-1 was compared in two groups using the Student's t-test for independent samples.

All results of thrombospondin-1 expression were analysed by the Student's t-test. Spearman's correlation coefficient was used to evaluate the relationship between parameters. Statistical significance was set at p < 0,05.

## Results

From 56 patients, five cases were ultimateley excluded, because the histological diagnosis was other than cystic ovarian endometriosis (2 teratomas and 3 haemorragic cysts). The presence and type of pelvic adherences, mean rAFS score and stages, and sizes of endometriomas were not statistically different between groups [15].

The mean thrombospondin-1 serum levels in group A was 256,69 pg/ml_+37,07 and in group B was 291,41 pg/ml + 35,59. In order to verify whether this observation could have been biased by the lack of control for several possible confounders, the mean thrombospondin-1 serum levels was adjusted with respect to gravidity, length of menses, infertility and BMI in a univariate general linear model [16]. Using this model, no significant difference was observed in mean thrombospondin-1 serum levels between two groups.

Serum thrombospondin-1 concentration did not correlate with the diameter of the endometriomas and the severity of the endometriosis, assessed according to revised AFS scores.

## Conclusion

The presence of ovarian cystic endometriosis is associated with pelvic pain in women suffering this disease [8]. On the other hand, angiogenic factors have been found increased in ovarian endometriomas [6]. Angiogenesis is related to vascularization. Therefore, a correlation between vascularization and the presence of pelvic pain might be assumed. Some studies assessing angiogenic activity in endometriosis have used either morphometric or inmunohistochemical techniques in endometriotic tissue [6,17-19]. Other studies have evaluated vascular activity measuring serum [16,20] or peritoneal fluid concentrations of angiogenic factors, such as VEGF [1,3]. Previously, some authors assessed that angiogenic factors are increased in the serum of patients with endometriosis [18] when compared with patients without endometriosis. Recently, Ohata has been demostrated that thrombospondin-1 serum levels were higher in patients with ovarian endometrioma than in patients without endometriosis [21,22].

Previously, we demonstrated for the first time that IL-8 and VEGF serum levels is not increased in patients diagnosed of ovarian endometriomas who presenting pelvic pain as compared with those who are asymptomatic. Some authors, have been demonstrated that expresion of TSP-1 is higher in endometriotic lesions and is associated to the extent of their vascularization.

In the present study, we analysed if thrombospondin-1 serum levels were correlated with ovarian endometrisosis and pelvic pain. We conclude that although thrombospondin-1 seems to play a key role in the local development of endometriotic lesions, the disease is not associated with a significant modulation in the levels of circulating thrombospondin-1 and the activity of endometriosis can not be monitored using serum levels.

Although recently studies have demonstrated that IL-8 and thrombospondin-1 serum level improve diagnostic reability of ovarian endometriosis we believe that the optimal serum marker should be used to monitoring the response of new antiangiogenic agents used in endometriosis treatment.

## Abbreviations

pg/ml: picograms/mililiter; VEGF: Vascular Endothelium Growth Factor; IL-8: Interleukin 8; TSP-1: Thrombospondin-1; VAS: Visual Analogic Scale; °C: Centrigrade degrees; BMI: Body Mass Index; rAFS scores and stages: revised American Fertility Society scores and stages.

## Competing interests

The authors declare that they have no competing interests.

## Authors' contributions

MGM, designed the study and wrote the paper. BO and PR reviewed the literature related and corrected all areas in the text including english language of the paper, covering this fields. JLA was responsible for the methodological and statistics corrections.
